# Implication of Intestinal Barrier Dysfunction in Gut Dysbiosis and Diseases

**DOI:** 10.3390/biomedicines10020289

**Published:** 2022-01-27

**Authors:** Carmine Stolfi, Claudia Maresca, Giovanni Monteleone, Federica Laudisi

**Affiliations:** 1 Department of Systems Medicine, University of Rome “Tor Vergata”, 00133 Rome, Italy; maresca.9595@gmail.com (C.M.); gi.monteleone@med.uniroma2.it (G.M.); 2 Division of Clinical Biochemistry and Clinical Molecular Biology, University of Rome “Tor Vergata”, 00133 Rome, Italy

**Keywords:** intestinal epithelial cells, mucosal barrier, microbiota, inflammatory bowel diseases, diet, mucus layer, aryl hydrocarbon receptor, cell commitment, junctional complexes, Paneth cells

## Abstract

The intestinal mucosal barrier, also referred to as intestinal barrier, is widely recognized as a critical player in gut homeostasis maintenance as it ensures the complex crosstalk between gut microbes (both commensals and pathogens) and the host immune system. Highly specialized epithelial cells constantly cope with several protective and harmful agents to maintain the multiple physiological functions of the barrier as well as its integrity. However, both genetic defects and environmental factors can break such equilibrium, thus promoting gut dysbiosis, dysregulated immune-inflammatory responses, and even the development of chronic pathological conditions. Here, we review and discuss the molecular and cellular pathways underlying intestinal barrier structural and functional homeostasis, focusing on potential alterations that may undermine this fine balance.

## 1. Introduction

**Figure 1:**
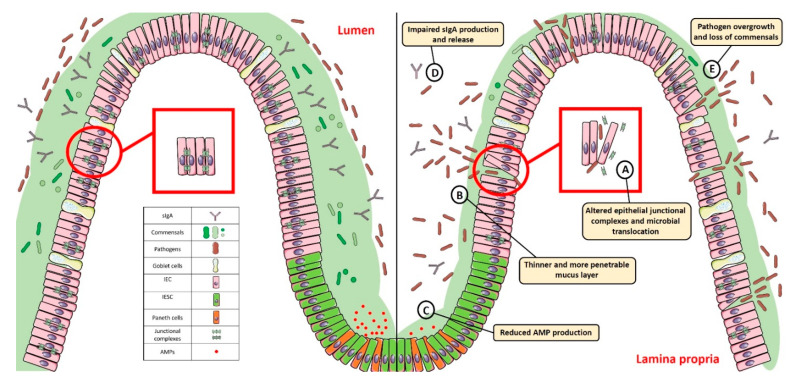
Gut homeostasis is established and maintained by the intestinal mucosal barrier. Alterations in its integrity and function, characterized by: (**A**) dysregulated junctional complexes, (**B**) thinner mucus layer, (**C**,**D**) reduced AMP and IgA production, and (**E**) pathogen overgrowth and penetration across the epithelial barrier, may perturb this fine balance and lead to gut dysbiosis. SIgA: Secretory Immunoglobulin A; AMP: antimicrobial peptide; IEC: intestinal epithelial cell; IESC: intestinal epithelial stem cell.

The intestinal mucosal barrier, also referred to as intestinal barrier, is a selectively permeable structure that grants the absorption of water, electrolytes, and essential dietary nutrients from the intestinal lumen into the circulation [1,2]. Apart from this role, the intestinal barrier mediates the crosstalk between commensal gut microbes and the host immunity and constitutes a first line of defence against intraluminal pathogenic antigens and potentially harmful microorganisms [1,2]. The intestinal barrier is composed of several elements that aid in its function as a physical and immunological defence boundary. These mainly include: (i) the outer mucus layer, encompassing the commensal gut microbiota, antimicrobial proteins (AMPs), and secretory immunoglobulin A (SIgA) molecules; (ii) the central single layer of specialized epithelial cells, derived from a pool of pluripotent stem cells at the base of the crypts that can be ultimately committed to goblet cells (which secrete mucins), Paneth cells (which synthesize antimicrobial peptides such as lysozyme and defensins), enteroendocrine cells (producing enteric hormones), enterocytes (absorbing water and nutrients), and Microfold cells, also referred to as M cells, (which are specialized for antigen sampling), following the up- or down-regulation of specific transcription factors; (iii) the inner lamina propria where cells from both innate (e.g., natural killer, neutrophils) and adaptive (e.g., T cells, B cells) immunity reside (Figure 1).

The ability to regulate the physiological processes occurring in the gut to keep internal states steady and balanced, also referred to as intestinal homeostasis, depends on complex interactions between the microbiota, the intestinal epithelium, and the host immune system. In particular, intestinal epithelial cells (IECs) act as frontline sensors for microbial encounters, and their hyporesponsiveness is ensured by the host innate immune system that can discriminate between signals derived from either commensal bacteria or pathogens [3,4,5]. Increasing evidence in germ-free mice highlighted the importance of commensal microbiome in maintaining gut homeostasis by providing protective, structural, and metabolic effects on the host mucosal surfaces [6,7]. For instance, commensals can release anti-microbial peptides, synthetize vitamins, contribute to ion adsorption and fermentation of non-digestible dietary residues, control epithelial cell differentiation, induce IgA secretion, and favour the immune system development [8,9]. Maintenance of such intestinal homeostasis also requires the structural integrity of the intestinal epithelium, which is ensured by junctional protein complexes (i.e., tight junctions, adherens junctions and desmosomes) that finely regulate intestinal permeability and seal adjacent epithelial cells [10] (Figure 1).

Dysfunction of the barrier physical integrity and/or an impaired function of the highly specialized cells composing the epithelial layer may lead to pathogen invasion and mucosal dysbiosis, resulting in a disruption of gut homeostasis that may ultimately trigger pathologic conditions, such as inflammatory bowel diseases (IBD), celiac disease, *Clostridioides difficile* infection (CDI), irritable bowel syndrome, colorectal cancer, type 1 diabetes, and obesity (Table 1) [11,12,13,14].

Here, we review and discuss the available experimental evidence about defects in epithelial barrier integrity and function and how they can compromise gut homeostasis, thus favouring microbial dysbiosis and disease development.

## 2. Breaking the Balance: Intestinal Barrier Dysfunction and Gut Dysbiosis

Both genetic defects and specific environmental factors are known to contribute to break the intestinal barrier balance and promote gut dysbiosis. In particular, impaired expression of genes related to cell commitment, junctional complexes, mucus production and secretion, Paneth cell activity, pathogen sensing, reactive oxygen species (ROS) production, xenobiotic response, and IgA secretion dramatically compromise intestinal epithelial barrier integrity and protective function (Table 2). Similarly, environmental factors—including bacterial infections; medication exposure (e.g., antibiotics) subsequent to pathogen infections or other diseases; and increased intake of high-fat compounds, sugars, and ethanol at the expense of fruits and vegetables—were reported to affect host microbiota composition and metabolic activities, leading to loss of commensals and overgrowth of pathogens (Table 3).

Such defects and factors, summarized in Table 2 and Table 3 respectively, are discussed below.

### 2.1. Impairment of Cell Commitment

**Figure 2:**
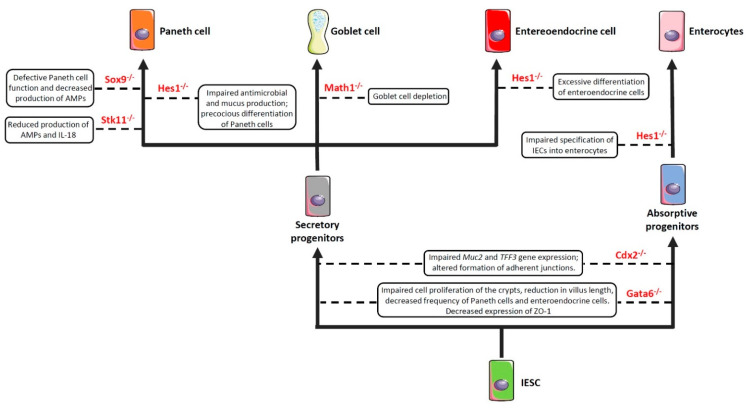
Impaired expression of genes encoding commitment-related transcription factors compromises epithelial cell differentiation and intestinal barrier function. Boxes enclose the effect/s of the knockdown of the genes depicted in red on the indicated cell commitment. *Abbreviations*: Sox9: SRY-Box Transcription Factor 9; Hes1: Hairy and enhancer of split 1; Stk11: Serine threonine kinase 11; Math1: Mouse atonal homolog 1; Cdx2: Caudal type homeobox 2; Gata6: GATA binding factor 6; Muc2: Mucin 2; TFF3: Trefoil factor 3; ZO-1: Zonula Occludens-1; AMPs: Antimicrobial peptides; IESC: Intestinal epithelial stem cells.

As previously anticipated, the intestinal barrier is characterized by a self-renewing epithelium, organized in crypts and villi, including both stem cells and differentiated cells. As the epithelial barrier has to deal with multiple physiologic activities, it requires different specialized cells, some of which are able to produce and secrete several molecules—such as antimicrobial peptides, mucins and hormones—and others that are able to adsorb water and nutrients. To achieve this goal, stem cells, after multiple transit-amplifying (TA) divisions, terminally differentiate into either secretory or absorptive lineages depending on the tightly regulated expression/inhibition of specific transcription factors [114,115]. For instance, the cell surface receptor Notch drives the cell commitment process by binding to the Notch ligands Deltalike (Dll) and Jagged families [116]. A cell expressing the Notch ligands will differentiate into a secretory cell (e.g., goblet cell, Paneth cell) upon expression of the *Atonal BHLH Transcription Factor 1* (*Atoh1*), whereas a neighbour cell expressing the activated Notch receptor will induce the expression of the target gene *hairy and enhancer of split 1* (*Hes1*)—a Atoh1 inhibitor—and will differentiate into an absorptive cell (e.g., enterocyte) [54]. Obviously, a dysregulated expression of the above-mentioned proteins definitely compromises the intestinal epithelial cell commitment and, in turn, the intestinal barrier integrity and function, as demonstrated by the association of several polymorphisms in genes encoding commitment-related transcription factors with intestinal barrier dysfunctions and intestinal inflammatory diseases (Figure 2) [51,52,53,54,55]. In this context, by employing genetically engineered mouse models, Guo and colleagues demonstrated that deficiency of the *Hes1* gene in IECs negatively affected antimicrobial peptides and mucus production, thus resulting in gut dysbiosis and inflammation [51]. Along the same line was the demonstration that mice deficient for *Math1* (also referred to as *Atoh1*) displayed complete abrogation of goblet cells in the intestine [54]. Similar results were observed in mice with conditional deletion of the *serine threonine kinase 11* (*Stk11*) gene, involved in the differentiation of stem cells into the secretory lineage cell types (e.g., goblet cells and Paneth cells), in IECs [55]. These animals displayed increased susceptibility to gut inflammation in association with reduced production of antimicrobial peptides and IL-18, as well as an uncontrolled expansion of colitogenic bacteria [55].

Another important transcription factor involved in the commitment of IECs is the *caudal type homeobox 2* (*Cdx2*) gene [117]. Indeed, CDX2 is a positive regulator of the *Muc2* and *Trefoil Factor 3* (*TFF3*) genes [56,118] involved in the production and stabilization of the mucus layer, respectively, and whose deficiency induces hypersensitivity to chemically-induced colitis (such as that induced by dextran sulfate sodium (DSS)) [15,56]. In addition, CDX2 controls cell-cell interactions and the expression of cadherins, which are important in the formation of the adherens junctions [119,120,121]. In support of this view is the evidence that mice bearing one non-functional *Cdx2* allele (*Cdx2*^+/−^ mice) displayed increased intestinal permeability and were more susceptible to the abrasive effect of DSS [117].

The GATA binding factor 6 (GATA6) is a zinc finger transcription factor that regulates cell proliferation, differentiation, and gene expression in several tissues [122]. For instance, GATA6 is involved in cell proliferation and differentiation along the gastrointestinal tract [57,58]. In particular, conditional deletion of *Gata6* in IECs resulted in impaired cell proliferation of the crypts, reduction in villus length, decreased frequency of Paneth cells and enteroendocrine cells, increased number of goblet-like cells, and dysregulated expression of enterocyte-related genes in the ileum [57]. Similar alterations were observed in the colon, where *Gata6* deficiency affected stem cell proliferation and differentiation into Paneth cells, enteroendocrine cells, and enterocytes [58]. Our study has recently demonstrated that conditional deletion of *Gata6* in the gut epithelium significantly affected intestinal barrier integrity, leading to decreased expression of the tight junction-related protein zonula occludens-1 (ZO-1), and resulting in increased paracellular permeability, microbial dysbiosis, and susceptibility to gut inflammation [59]. Interestingly, we also reported a decreased expression of GATA6 in the intestinal epithelium of IBD patients, thus suggesting that a reduced expression of this transcription factor may contribute to intestinal barrier dysfunction in these subjects [59]. In the intestinal epithelium, defects in Paneth cell function—and the consequent decrease in the antimicrobial peptide production—may also result from the deletion of *Sox9* [60]. Indeed, by generating mice that harbored a conditional *Sox9* gene and a Villin-Cre transgene, Mori-Akiyama et al. reported that lack of *Sox9* expression in the intestinal epithelium of *Sox9*^fl/fl^ Villin-Cre^+^ mice resulted in the complete absence of differentiated Paneth cells, although the differentiation of other intestinal epithelial cell subsets (e.g., goblet cells, enterocytes) was not affected. Moreover, *Sox9* deficiency also lead to crypt enlargement, a marked increase in cell proliferation throughout the crypts, as well as a replacement of the Paneth cells by proliferating epithelial cells [60]. More recently, by employing the same conditional mouse model, Riba and colleagues showed that *Sox9* deletion in the intestinal epithelium reduced lysozyme production. This effect resulted in significant microbial dysbiosis, characterized by *E. coli* overgrowth and ultimately leading to visceral hypersensitivity [61].

### 2.2. Impairment of Epithelial Junctional Complexes

The intestinal epithelial barrier’s main function is to protect the host from luminal antigens, pathogens, and toxins, while allowing selective permeability to water, nutrients, and electrolytes. In particular, transcellular permeability, involved in solute transport through the epithelial cells, is mediated by selective transporters for amino acids, electrolytes, short chain fatty acids, and sugars [123]. Paracellular permeability, instead, occurs through intercellular junctional complexes encompassing adherens junctions (AJs), tight junctions (TJs), and desmosomes [124,125]. These transmembrane proteins, localized both at the apical-lateral membrane junction and along the lateral membrane, mediate the contact between adjacent IECs, thus sealing the intracellular spaces. The AJs (e.g., catenins, cadherins) and desmosomes (e.g., desmoglein, desmocollins) regulate the mechanical linkage of adjacent cells, while the TJs (e.g., ZO-1, claudin-2, occludins, Junctional Adhesion Molecule) form an apical junctional complex that seals the intercellular space and modulates selective paracellular permeability [124,125,126,127,128].

Alterations in the formation/distribution of the intercellular junctional complexes, which may occur in men with specific genetic susceptibilities, as well as in response to dietary factors and bacterial infections, may result in intestinal epithelial barrier breakdown and translocation of the luminal content into the lamina propria, leading to gut dysbiosis, uncontrolled immune/inflammatory responses, and, ultimately, pathological conditions [10].

For instance, gliadin (a glycoprotein representing the major component of wheat gluten) has been reported to deeply affect the expression and distribution of several junctional complexes in the small intestine of celiac patients by binding to CXC motif receptor 3 (CXCR3) on epithelial cells [20]. This interaction induces the release of zonulin, a human protein analogue of the Zonula occludens toxin (ZOT) from *Vibrio cholerae*, through the recruitment of Myeloid differentiation primary response (MyD)-88 [20,21,129,130]. Increased levels of zonulin were detected in the intestinal tissues taken from celiac disease patients during the acute phase compared to those taken from healthy controls. Once released, Zonulin leads to transactivation of EGF receptor (EGFR) via proteinase-activated receptor 2 (PAR2) activation in the intestinal epithelium and subsequent tight junction disassembly [20,21,129,130].

The impairment of the epithelial junctional complexes importantly contributes to the development of other chronic inflammatory conditions, such as IBD. For example, increased expression of claudin-2, as well as impaired expression and redistribution of claudin-5, -8 and occludin were reported in Crohn’s disease patients, leading to increased intestinal permeability and bacterial translocation [131]. A similar severe condition was described also in the colonic mucosa of patients with ulcerative colitis, in association with the dysregulated expression of occludin, ZO-1, claudin-1, JAM, beta-catenin, E-cadherin, and the consequent transepithelial migration of neutrophils [132].

The above-mentioned chronic inflammatory conditions importantly contribute to the development and progress of colorectal carcinogenesis. Interestingly, increased expression of claudin-1 and claudin-2 was found to correlate with inflammatory activity, IBD-associated dysplasia, and sporadic adenomas [36]. Similarly, Dhawan et al. observed that claudin-1 expression was increased in colon carcinomas and metastatic lesions and played a key role for tumorigenesis and invasiveness of colonic epithelial cells [35]. Claudin-2 was also reported to be increased in tissues taken from CRC and IBD-associated CRC patients and to promote and sustain cell proliferation and tumor growth in cultured cells and experimental models [133].

Dysfunctions of the epithelial junctional complexes and the consequent increase of intestinal permeability and gut dysbiosis correlate with the development and progression of other pathological conditions. In particular, increased intestinal permeability was seen to precede and/or to be an early biomarker of diabetes development in patients, as well as in experimental models of the disease [45,134,135]. Moreover, increased serum levels of zonulin, in association with altered intestinal permeability, were described in a subgroup of patients with type 1 diabetes and their first-degree relatives, suggesting this molecule as a valid early biomarker of disease development [49].

Animal models employing genetically engineered mice have helped to better understand the link between junctional complex dysregulation and the development of dysbiosis and pathologic conditions. In this context, Laukoetter et al. reported a role for Junctional Adhesion Molecule (JAM)-A, a TJ component contributing to the control of barrier function and leukocyte migration, in regulating intestinal permeability and inflammation in vivo [62]. Indeed, despite showing normal epithelial architecture, *JAM-A* knockout mice developed low-grade colonic inflammation (characterized by enhanced polymorphonuclear leukocyte infiltration and large lymphoid aggregates not seen in sham mice) [62]. Barrier function experiments revealed increased mucosal permeability, as indicated by enhanced dextran flux, and decreased transepithelial electrical resistance in *JAM-A* knockout mice compared to wild-type control mice [62]. Consistently, *JAM-A* deficiency increased the permeability of in vitro monolayers derived from the human colonic epithelial cell line SK-CO15 compared with control. Moreover, *JAM-A* deficient mice were more susceptible to the DSS-driven experimental colitis compared to controls, although the colonic mucosa showed less injury and increased epithelial proliferation [62]. Analyses of other TJ-related proteins showed increased expression of claudin-10 and -15, both of which tune TJ barrier function by the formation of ion-selective pores, following *JAM-A* knockdown in the colonic mucosa of mice and in SK-CO15 cell monolayers [62].

In a later article, Wada and colleagues reported that mice with the double knockdown of *claudin-2* (*Cldn-2*) and *claudin*-15 (*Cldn-15*) genes had impaired paracellular Na^+^ flow and subsequent malnutrition, leading to infant death [63].

By employing cultured epithelial cells and an intestinal epithelial-specific knockout mouse (that is, *Tjp1*^fl/fl^ Villin-Cre^+^ mouse), Odenwald and co-workers showed that the TJ scaffolding protein ZO-1 was essential for development of unified apical surfaces in vitro and in vivo. In detail, conditional deletion of ZO-1 in IECs of *Tjp1*^fl/fl^ Villin-Cre^+^ mice did not significantly alter crypt-villus architecture, whereas it affected apical tissue continuity, which is by characterized apical surface brush border membrane, and the presence of crevasses at intercellular junctions between enterocytes, likely by modulating actomyosin contraction and membrane traffic [64]. Recently, Kuo and colleagues reported decreased ZO-1 expression, both at RNA and protein level, in intestinal mucosal biopsies isolated from IBD patients as compared with those isolated from healthy controls [65]. Loss of ZO-1 expression in epithelial cells in *Tjp1*^fl/fl^ Villin-Cre^+^ mice did not promote spontaneous disease, but it exacerbated tissue damage and weight loss during experimental colitis, as well as delayed the mucosal healing [65]. The authors also reported that ZO-1 is critically involved in the cell division phase upon damage. In particular, by associating with the centriole and mitotic spindle, ZO-1 contributed to both Wnt–β-catenin signaling and mitotic spindle orientation, suggesting that ZO-1 may actively contribute to the intestinal epithelial barrier restoration [65]. In line with these observations, we recently found that loss of *Gata6* expression in IECs of genetically engineered mice resulted in increased intestinal permeability, gut dysbiosis, and microbial-driven intestinal inflammation. These effects were associated with decreased ZO-1 expression and epithelial damage both in the ileum and colon. Experiments in cultured cells suggested that ZO-1 expression could be directly modulated by GATA6 [59]. Recently, Marchelletta and colleagues reported that the impaired function of T cell protein tyrosine phosphatase (TCPTP), encoded by the *protein tyrosine phosphatase non-receptor type 2* (*PTPN2*) gene, contributed to the epithelial tight junction protein remodeling and increased intestinal permeability [66]. In particular, *Tcptp*-deficient mice showed increased claudin-2 expression, intestinal permeability, and inflammatory cytokine production [66]. In detail, TCPTP was able to maintain the localization of ZO-1 and occludin at apical tight junctions, as well as to modulate the turn-over of claudin-2, a cation pore-forming transmembrane protein, by upregulating the serine metalloproteinase matriptase, which promoted claudin-2 proteosomal degradation [66].

Apart from defects in the above-mentioned epithelial junction-related molecules, several dietary factors may contribute to increase intestinal permeability and trigger/amplify pathologic conditions [136]. A good example in this regard is given by gluten, which, in addition to its well-known detrimental effects on barrier integrity and TJ protein activity in celiac disease, can actively promote dysregulation of intestinal barrier function in non-celiac patients. Of note, mice exposed to a gluten-rich diet showed alterations in adherent junctions and desmosomes, resulting in increased intestinal permeability and susceptibility to DSS-driven experimental colitis [95].

Glucose and fructose are additional macronutrients found to trigger TJ and AJ protein dysfunction, thus promoting changes in microbiota composition, increased susceptibility to pathogen infection, as well as metabolic syndrome [44]. In mouse experimental models, uncontrolled metabolism of fructose in the liver and in the small intestine, due to the excessive delivery of this sugar (15% in water for 3 weeks), induced the transcriptional expression of fructokinase (a protein involved in fructose metabolism), TJ alterations, energy depletion, oxidative stress, and chronic inflammation [96]. On the other hand, mice deficient of the fructokinase isoforms A and C (*KHK-A*, *KHK-C*) were protected from such detrimental effects. Notably, loss of KHK-A function only did not prevent alterations in TJs, thus suggesting that intestinal barrier impairment was mainly mediated by KHK-C activity [96].

Detrimental effects of dietary fats on the epithelial junctional complexes have been also reported by several studies. In particular, mice exposed to a high-fat diet for 3, 11, and 22 weeks showed induction of endoplasmic reticulum (ER) stress in IECs, as well as an impairment of Claudin-1 expression and mucus barrier, with the consequent increase of endotoxin serum levels and gut dysbiosis [97]. Similarly, Devkota and colleagues demonstrated that the increased availability of taurocholic bile acid, due to the consumption of a diet high in saturated (milk derived)-fat, promoted the expansion of the low abundance pathobiont *Bilophila wadsworthia* (a member of the *Deltaproteobacteria*), which, in turn, was able to impair intestinal barrier integrity in genetically susceptible *Il-10*^−/−^ mice due to its sulphite-reducing activity [98]. Another dietary habit found to affect TJ activity is ethanol consumption. Exposure to non-cytotoxic doses of ethanol (as those detected in the blood of moderate drinkers) impaired paracellular permeability in vitro due to alterations in ZO-1 and occludin localization [99,100].

Both localization and activity of epithelial junctional complexes can also be affected by pathogen invasion and toxin secretion. For instance, *Salmonella typhimurium* was found to up-regulate the colonic expression of the leaky protein claudin-2, which plays an opposite role in the modulation of intestinal permeability compared to other TJ proteins involved in barrier maintenance, thus facilitating bacterial invasion [108]. *Vibrio cholerae*, instead, was reported to target the intestinal epithelial barrier by producing the zonula occludens toxin (ZOT), which transiently affects the paracellular permeability in the small intestine by opening TJs through a protein kinase C-dependent actin reorganization [109,110].

On its side, antibiotic treatment dramatically influences intestinal permeability by compromising host microbial ecology. In particular, mice exposed to antibiotics for 2 weeks developed mucosal dysbiosis characterized by decreased production of short-chain fatty acids, such as butyrate (known to sustain barrier function and integrity), by commensals [112]. Moreover, antibiotic treatment hampered intestinal TJ function and increased intestinal permeability by reducing the expression of ZO-1, occluding, and claudin-1 [112]. Similar results were obtained in antibiotic-treated germ-free mice, which presented altered microvilli morphology and reduced rate of intestinal epithelial cell turnover compared to sham mice [113]. Altogether, these results indicate a key role for commensal microbiota in preserving epithelial junctional complexes and gut barrier integrity, highlighting a possible detrimental effect of antibiotic exposure on such a fine balance.

### 2.3. Thinning/Depletion of the Mucus Layer

Goblet cells are specialized IECs able to synthetize and secrete mucin proteins into the lumen. Mucin proteins are pivotal in creating a protective mucus layer acting against pathogens, chemicals, and mechanical stress in order to maintain gut homeostasis and protect the inner mucosal surface [137]. The mucus layer, mainly composed of water, electrolytes, lipids, and glycosylated mucins [138], represents an important source of antimicrobial peptides and immunoglobulins and can directly interact with commensals, providing nutrients and attachment sites depending on the mucin glycosylation profile [139].

Mucolytic bacteria (e.g., *Akkermansia muciniphila, Bacteroides thetaiotaomicron, Ruminococcus gnavus, Ruminococcus torques*) represent an important class of commensals as they are able to digest glycans (from dietary fibers) and mucins through glycosidase enzymes, and to produce, in turn, short chain fatty acids (such as acetate and butyrate) acting as energy source for colonocytes and contributing to protect the intestinal barrier integrity [139]. However, the fine balance between goblet cell-mediated replenishment of mucus and its degradation by commensals can be affected by a fiber-deprived diet, as indicated by the fact that mice subjected to intermittent dietary fiber deprivation presented a thinner mucus layer due to O-linked glycan digestion by the fiber-deprived microbiota [101]. Thus, enrichment in mucus-degrading bacteria may impair the mucus layer thickness and viscosity and promote enteric pathogens adherence and penetration, ultimately causing gut dysbiosis and chronic intestinal inflammation [67,101]. These pathological alterations were observed in the *Winnie* murine model of spontaneous colitis, characterized by a missense mutation in the *Muc2* gene [16,67]. The phenotype of *Winnie* mice was characterized by altered mucus production as early as 4 weeks of age, with ensuing intestinal barrier dysfunction, gut dysbiosis, and inflammation [16,67]. In particular, impaired *Muc2* expression affected the number of goblet cells, which underwent unresolved ER stress and accumulation of mucin precursors [16,67]. All these processes were associated with apoptotic cell death, increased intestinal permeability, pathogen penetration into the inner mucus layer, and adherence to epithelial cells, as well as bacterial translocation into the lamina propria [16,67]. The subsequent uncontrolled immune response towards pathogens (e.g., enhanced dendritic cell activation, T-helper cytokine production) promoted chronic intestinal inflammation and gut dysbiosis, characterized by the outgrowth of *Bacteroidetes* and *Verrucomicrobia* (such as *Akkermansia muciniphila*) [67,68].

Impaired mucus layer integrity can also depend on mutations in the *Gfi1* gene. Gfi1 functions downstream of *Math1* in the intestinal epithelium and encodes molecules involved in the stem cell differentiation into the different secretory cell lineages [69]. In particular, *Gfi1*-deficient mice displayed alterations in terminal differentiation and morphology of goblet cells and Paneth cells, together with accumulation of immature secretory progenitors, as well as a decrease in mucin and antimicrobial peptide release [69]. Recently, the Foxo1 trascription factor was described to be critically involved in mucin granule release through autophagy [70]. In particular, *Foxo1*^fl/fl^ Villin-Cre^+^ mice showed impaired mucus layer formation and subsequent dysbiosis, resulting in disrupted intestinal barrier integrity and enhanced susceptibility to infection and tissue inflammation. Moreover, *Foxo1* deficiency in IECs resulted in the overgrowth of mucin-degrading bacteria and a decrease of short-chain fatty acid-producing microbial species, which further affected the intestinal barrier function [70].

In addition to mucus production and degradation, gut microbiota composition is able to influence the mucus properties. In this regard, Jakobsson and colleagues reported that the mucus layer of germ-free mice was characterized by a higher mucus penetrability as compared to conventional mice [111]. Moreover, mice with identical genetic background, but hosted in two rooms of the same specific pathogen-free animal facility, showed different mucus properties, evidenced by the fact that one colony had an impenetrable inner mucus layer, whereas the other showed opposite features [111]. The authors suggested that these differences relied on changes in the gut microbiota composition as the different mucus phenotypes were acquired by germ-free mice upon faecal microbiota transplantation [111]. In particular, mice with an impenetrable inner mucus layer showed increased frequency of the *Erysipelotrichi* class, whereas *Proteobacteria* and *TM7* expanded in mice with more penetrable mucus [111]. Hence, even genetically identical animals housed in the same facility may have distinct microbiotas and barrier structures [111].

Taken together, these results highlight the mutualistic effects between the gut microbial community and the mucus layer and their consequences on intestinal barrier integrity and function.

### 2.4. Paneth Cell Dysfunction

Intestinal epithelial cells include Paneth cells, a particular group of secretory cells located at the base of the crypts of Lieberkühn in the small intestine. As previously described, Paneth cells, together with goblet cells and enteroendocrine cells, originate from a common progenitor that expresses the *Math1* gene [54]. Further differentiation of the secretory lineage into Paneth cells and goblet cells requires additional key transcription factors, such as: (1) Gfi1, a zinc-finger protein family member that functions downstream of Math1; (2) Sox9, which controls an early step of Paneth cell differentiation; (3) Fz5, which is crucial for the late step of cell commitment toward the Paneth cell phenotype; and (4) Cdk5rap3, which is involved in both fate decision and cell development [60]. Once differentiated, Paneth cells migrate to the base of crypts, instead of out of crypts onto adjacent villi. These specialized epithelial cells are able to produce and secrete granules enclosing AMPs (e.g., α-defensin, Reg3 lectins, lysozyme, and secretory phospholipase A2 isotype II) that shape the composition of commensals and protect the host from pathogen colonization [140,141]. Interestingly, mice with deficiency for vitamin D and exposed to high-fat diet showed decreased expression of Paneth cell-specific alpha-defensins, including α-defensin 5 (DEFA5), Matrix metalloproteinase 7 (MMP7), and tight junction genes [102]. Such defects resulted in enhanced gut permeability, microbial translocation, and consequent gut dysbiosis, as well as chronic inflammation and metabolic syndrome [102]. In addition, an increased fraction of abnormal Paneth cells that exhibit ER stress and accumulate reduced-form α-defensins were observed in SAMP1/YitFc mice, representing an animal model of Crohn’s disease-like ileitis [103]. In particular, secretion of misfolded α-defensins resulted in mucosal dysbiosis characterized by loss of *Lachnospiraceae* and *Ruminococcaceae* and increased abundance of *Bacteroidaceae* and *Rikenellaceae* [103]. Interestingly, administration of reduced-form α-defensins to wild-type mice induced similar microbial alterations, thus suggesting that Paneth cell activity was crucial to keep/ensure gut homeostasis [103].

Paneth cell dysfunction and consequent intestinal dysbiosis may also derive from the presence of risk alleles that commonly associate with chronic inflammatory pathologies, such as Crohn’s disease. In particular, the *Nod2* gene encodes the nucleotide-binding oligomerization domain 2 (NOD2), a cytoplasmic muramyl dipeptide receptor able to recognize both Gram-positive and negative pathogens. NOD2 is highly expressed by Paneth cells and impaired NOD2 expression compromises α-defensins secretion, affecting gut homeostasis and promoting increased susceptibility to infections [71,72]. Other genetic polymorphisms associated with Paneth cell dysfunction (such as *Xbp1* and *Atg16l1* polymorphisms) are related to chronic ER stress and impaired autophagy in response to viral infection, leading to decreased AMP secretion [73,74,75]. Moreover, susceptibility polymorphisms in the promoter region of the *Tcf4* gene also associate with Paneth cell dysfunction and Crohn’s disease development [76,77].

Paneth cell activity can also be hampered by dietary compounds. For instance, a high-fat diet is able to enhance the secretion of bile acids which, in turn, promote the upregulation of the G protein-coupled bile acid receptor (TGR5) on Paneth cell membrane [104]. The interaction between bile acids and TGR5 resulted in decreased expression of anti-microbial peptide-related genes (such as *α-defensin 5* and *6*) and induction of ER stress, autophagy, and DNA damage. Moreover, gut microbiota composition was significantly affected, with reduced abundance in *Firmicutes* and *Lactobacillaceae*, whereas *Verrucomicrobiaceae*, *Akkermansia muciniphila*, *Clostridium XIVa*, *Ruminococcaceae*, and *Lachnospiraceae* resulted to be increased [104]. Mice exposed to high-fat diet and orally treated with Cholestyramine (a bile acid sequestrant) or 4-Phenylbutyric acid (a ER stress inhibitor) prevented these changes as both molecules reduced serum bile acid levels and decreased TGR5 expression on Paneth cells without altering microbiota composition [104]. Finally, a Western diet has been suggested to indirectly impact on Paneth cell activity through the *Clostridium*-mediated conversion of the secondary bile acid deoxycholic acid. In detail, increased level of deoxycholic acid in the ileum induced excessive activation of the farnesoid X receptor (FXR) and type I interferon (IFN) signalling pathways, ultimately leading to Paneth cell defects [105].

Thus, impaired Paneth cell activity, induced by bile acid toxicity following a high-fat diet or other triggers, can deeply impact on host microbial composition and gut homeostasis, ultimately leading to the onset and development of gut dysbiosis and several chronic pathologic conditions.

### 2.5. Impairment of Microbial Sensing by Pattern Recognition Receptors (PRRs)

The efficient interaction/crosstalk between host and commensals across the intestinal epithelial barrier involves several inducible mechanisms that are able to discriminate endogenous and exogenous luminal antigens and to modulate the host immune response accordingly. These mechanisms require the presence on epithelial cells of specific receptors termed “pattern recognition receptors” (PRRs), which drive the sensing of pathogens and the consequent initiation of innate inflammatory immune responses, while maintaining an immune tolerance towards resident gut microbiota [142].

Toll-like receptors (TLRs) represent an important class of microbial-induced proteins expressed in different cell compartments including cells of the intestinal lining (e.g., stem cells, enterocytes), which are able to recognize microbial-related molecules—such as flagellin and lipopolysaccharide (LPS)—as well as single- and double-stranded RNAs. Once activated, TLRs trigger specific signalling pathways, ultimately inducing the expression/activation of transcription factors (e.g., NF-kB, AP-1, IRF3) involved in the orchestration of pro-inflammatory responses [143].

TLR-mediated pathways play an important role in the regulation of intestinal barrier function and integrity. For instance, the activation of the TLR4/MyD88 signal transduction pathway by LPS was seen to increase the intestinal permeability by promoting IL-1 receptor-associated kinase (IRAK)-4 function and the phosphorylation of transforming growth factor-β–activating kinase (TAK)-1, followed by the activation of the canonical NF-kB pathway and the up-regulation of myosin light chain kinase (MLCK) [144]. Deficiency in MyD88 signalling increased intestinal epithelial cell proliferation, whereas activation of TLR4 by LPS triggered apoptotic cell death in murine intestinal organoids [78]. Further, TLRs actively contribute to the secretion of both mucus and antimicrobial peptides, IgA class switching, the expression of polymeric immunoglobulin receptor, translocation of ZO-1 and occludin to the tight junctions, as well as to the expression of nicotinamide-adenine dinucleotide phosphate (NADPH) oxidase and release of ROS [145,146,147,148].

Increasing evidence suggests that TLR function may be critically involved in the intestinal epithelial restitution. Indeed, TLR activity in enterocytes, goblet cells, and mesenchymal stem cells induces the expression of molecules (e.g., trefoil factor 3, prostaglandin E2) and downstream pathways (e.g., Wnt–β-catenin), promoting the proliferation of enterocytes in the crypts adjacent to the wound [149]. On the other hand, TLR function is regulated by specific molecules (e.g., IRAK3, SIGIRR), and impairment of this control system results in excessive TLR activity and consequent gut dysbiosis and detrimental inflammatory response [150]. Thus, it is clear that TLR activation has to be tightly regulated in order to prevent excessive/impaired epithelial TLR signalling, which could compromise microbial–host interaction and lead to inefficient pathogen clearance, increased intestinal permeability, gut dysbiosis, and chronic inflammation. Another PRR significantly contributing to gut homeostasis is NOD2, a cytosolic receptor expressed by both epithelial (e.g., Paneth cells, stem cells) and immune cells. Loss-of-function mutations in *Nod2* gene associate with increased susceptibility to Crohn’s disease due to impaired pathogen clearance by dendritic cells [79,80,81]. However, NOD2 function in epithelial cells is also crucial in order to maintain microbial ecology. In particular, upon sensing the bacterial muramyl dipeptide, N-acetylmuramyl-L-alanyl-D-isoglutamine (MDP) present in Gram-positive and -negative bacteria, NOD2 is able to trigger host defences through the production of antimicrobial peptides, cytokines, mucins, and the activation of immune responses, thus ensuring a balanced host-microbial crosstalk [151,152]. Given that, *Nod2*-deficient mice showed a decreased antimicrobial activity of Paneth cells and enhanced intestinal colonization by pathogens that could easily penetrate through the intestinal barrier and induce gut dysbiosis [82]. In addition to the above-mentioned functions, NOD2 exerts a protective effect against oxidative stress-mediated cell death on stem cells and sustains epithelial regeneration [83], thus contributing to the intestinal barrier integrity.

### 2.6. Modulation of Epithelial Oxidative Burst

TLR ligands are critical actors in the defence system against luminal pathogens as they promote the transcription of NADPH oxidase-related genes and the production/release of ROS by phagocytes in the lamina propria. In addition, ROS can be produced by IECs through the activity of the NADPH oxidases DUOX2 and NOX1 [153,154]. The former, in particular, is expressed at the apical membrane of enterocytes and dimerizes with DUOXA2 for maturation and cell membrane trafficking [153,154]. The main function of DUOX2 is to protect the host mucosal surfaces by releasing hydrogen peroxide in response to pathogens (e.g., *Salmonella typhimurium*, *Listeria monocytogenes*, *Campylobacter jejun*), thus contributing to the maintenance of gut homeostasis [153,155,156,157]. Given that, loss of DUOX2 activity results in enhanced pathogen translocation to host lymphatic tissue and activation of compensatory defence mechanisms [84]. Interestingly, Grasberger and colleagues identified a significant association between DUOX2 loss-of-function variants and IL-17C induction in IBD mucosal biopsies in response to Gram-negative bacteria, suggesting that DUOX2 variants may increase the risk of developing IBD [84]. However, it is worth underlining that chronic activation of DUOX2 in the inflamed tissues of IBD patients may sustain harmful inflammatory responses [158,159]. In this context, DUOX2 expression was not limited to apical surface of epithelial cells, but widely expressed along the crypt epithelium of IBD patients [158]. Moreover, enhanced DUOX2 expression in inflamed intestinal tissue associated with gut dysbiosis is characterized by *Proteobacteria* expansion in Crohn’s disease patients [160]. Altogether, these evidences suggest that a dysregulated DUOX2 function may be considered a sensitive marker of gut dysbiosis and intestinal inflammation in IBD patients [161].

During an infection process, DUOX2 activity can be modulated by another intestinal epithelium NADPH oxidase, namely NADPH Oxidase 1 (NOX1) [85]. In particular, lack of Cyba protein, which normally dimerizes with NOX1 to form a superoxide-generating NADPH oxidase, was seen to compromise DUOX2 activity in response to intestinal infection by *Citrobacter rodentium* in mice [85]. Interestingly, loss of mucosal NOX1 function did not exacerbate intestinal inflammation by *Citrobacter rodentium* as several commensal, including *Lactobacilli* such as *Lactobacillus leuteri and Lactobacillus murinus*, started producing hydrogen peroxide and downregulating *C. rodentium*-related virulence factors to ensure host protection and gut homeostasis [85]. Both NOX1 and DUOX2 function can be also triggered by TLR-4 expressed on IECs [145]. In this regard, using *Tlr-4*^fl/fl^-Villin-Cre^+^ mice, Burgueño and colleagues observed that dysregulated TLR-4 signalling in IECs led to NOX1 and DUOX2 overexpression [145]. Further, dysregulated hydrogen peroxide synthesis altered gut microbiota and induced increased susceptibility to dysplasia and colon tumorigenesis once transplanted into recipient germ-free mice [145]. Hence, NOX1 and DUOX2 activity have to be tightly regulated in order to ensure pathogen clearance and prevent mucosal dysbiosis, as well as harmful intestinal inflammatory responses.

### 2.7. Modulation of Xenobiotic Receptors

Among their functions, IECs can also provide protection against xenobiotic substances (e.g., environmental pollutants, chemicals, drugs) thanks to a detoxification system encompassing specific enzymes involved in the elimination of toxic compounds from the host [162]. These detoxification enzymes are regulated by specific transcription factors, such as the nuclear receptor pregnane X receptor (PXR) and the drug receptor aryl hydrocarbon receptor (AhR), which are mainly expressed in the liver, small intestine, and colon [163,164]. Accumulating evidence suggests that these receptors are actively involved in the modulation of several physiologic functions (e.g., cell proliferation, cell death, inflammatory immune response) by recognizing food components and endogenous ligands [165,166]. In particular, PXR can regulate the expression of drug-metabolizing and drug-transporter enzymes, such as UDP glucuronosyltransferases (UGTs), glutathione S-transferases (GSTs), the cytochrome P450 (CYP) family, the multidrug resistance protein 1 (MDR1), and the multidrug-resistant associated proteins (MRPs) [167]. PXR is also a critical regulator of mucosal surfaces, known to recognize several endobiotic compounds (e.g., bilirubin, bile acids) [168]. PXR was seen to exert anti-inflammatory functions by preventing Iκ-Bα degradation, thus limiting the activity of the transcription factor NF-κB [169,170]. Moreover, PXR actively contributes to the intestinal barrier integrity and function. Indeed, *Pxr*-deficient mice showed increased intestinal permeability due to a reduction in ZO-1 and E-cadherin expression, as well as an increase in claudin-2 levels [86]. The dysregulated TLR4-NF-κB signalling pathway derived from the absence of PXR expression resulted in TNF-α over-production that further contributed to the increased paracellular transport across the epithelial barrier [86]. In detail, TNF-α induced ZO-1 relocalization through the upregulation of Myosin Light Chain Kinase (MLCK) expression, which, in turn, phosphorylates myosin II regulatory light chain (MLC), a protein involved in the junctional complex arrangement. PXR, instead, was seen to limit the MLCK upregulation by targeting NF-κB activation [165].

Moreover, PXR is also able to limit the phosphorylation and activation of C-jun N-terminal kinase (JNK) 1/2 by inducing the expression of the JNK1/2 inhibitory molecule GADD45β (growth arrest and DNA damage inducible 45β) [171]. Terc and colleagues also reported that PXR activation can mediate the intestinal epithelial cell migration and proliferation, as well as the repair of the intestinal barrier by inducing p38 mitogen-activated protein kinase (MAPK) activation following experimental colitis [165]. Decreased levels of PXR were observed in the inflamed tissue of IBD patients and polymorphisms of the PXR-encoding gene *Nr1I2,* which is significantly associated with increased susceptibility to disease [87,88].

In conclusion, PXR is a critical player in gut homeostasis maintenance and, for this reason, it should be considered a potential therapeutic target to manage chronic intestinal inflammation, gut dysbiosis, and epithelial damage.

Aryl hydrocarbon receptor (AhR) is a member of the basic helix–loop–helix (bHLH)–PAS family of transcription factors that act as environmental sensors (e.g., circadian rhythm, hypoxia, xenobiotic response) [172,173]. Aryl hydrocarbon receptor expression was originally characterized in the intestinal immune cells (e.g., Th17 cells, intraepithelial lymphocytes) where AhR-dependent signalling was crucial for immune cell survival and functional activity [174]. However, increasing evidence confirmed that AhR is also widely expressed in the intestinal microenvironment by non-hematopoietic cells (e.g., IECs) and its activation by natural ligands, such as dietary and microbial metabolites, results in the maintenance of gut homeostasis at the barrier sites (e.g., lung and gut) [175,176]. In particular, AhR deficiency in IECs causes increased susceptibility to the intestinal infection despite a normal immune compartment. Moreover, mice with dysregulated AhR expression in the epithelium showed dysfunctional barrier, characterized by reduced mucus production and impaired TJs, as well as chronic low-grade inflammation and crypt hyperplasia [89,90,91]. AhR was also seen to take part in the modulation of crypt stem cell proliferation, wound healing processes, and cell commitment. In particular, *Ahr*^fl/fl^-Villin-Cre^+^ mice showed impaired resistance against *Citrobacter rodentium* infection characterized by bacterial dissemination in the liver and in the spleen, increased epithelial damage, and decreased expression of *Muc2* and *Car4* genes, thus confirming a crucial role of AhR in the maintenance of gut homeostasis [90]. Furthermore, mice exposed to AhR ligand-free diet (e.g., high fat diet) developed gut dysbiosis, characterized by higher susceptibility to experimental colitis and overgrowth of *Erysipelotrichaceae* family compared to mice receiving the dietary AhR ligand indole-3-carbinol (I3C) [106,107]. Another important source of AhR natural ligands is represented by commensal microbiota. For example, the induction of AhR transcriptional targets was observed in IECs upon challenge with butyrate derived from *Proteobacteria*, *Firmicutes*, and *Fusobacteria,* and in part from *Actinobacteria* [177]. Overall, these observations indicate AhR activation in IECs by either xenobiotic compounds or natural ligands as a crucial step to ensure barrier integrity and host microbial ecology.

### 2.8. Impairment of Secretory IgA

Immunoglobulin A (IgA), the major immunoglobulin isotype secreted at the mucosal surfaces, provides the first line of defence against pathogens and toxins [178]. Maternal milk-derived secretory immunoglobulin A (SIgA) can mediate the protection at mucosal surfaces in neonates [179]. Moreover, SIgA is crucial in the regulation of host immune homeostasis as it can shape the composition and function of gut microbiota and promote bacteria–mucus and bacteria–bacteria interactions, leading to the release of metabolites enforcing mucosal barrier functions [180,181]. SIgA is mainly produced by plasma cells in the lamina propria, transported across the IECs through a transcytosis mechanism mediated by poly Ig receptor (pIgR), and released into the lumen in order to neutralize invading pathogens and related products via different mechanisms [182,183]. SIgA can modulate gut homeostasis by binding and excluding invading pathogens from the mucosal surface through a process called “agglutination”, thus preventing them from breaching the epithelial barrier and limiting consequent unwanted immune responses (immune exclusion) [184]. Moreover, SIgA are able to intercept incoming antigens intracellulary, thus generating immune complexes that are then transported by pIgR across the epithelial cells in order to be cleared [179,184]. Finally, SIgA immune complexes can be internalized by microfold cells (M cells) in the mucosal-associated lymphoid tissue and recognized by tolerogenic DCs, which, in turn, may secrete IL-10 and TGF-β cytokines and promote Foxp3^+^ regulatory T cells expansion, thus limiting potential detrimental inflammatory immune responses [185,186].

Given the above-mentioned functions, it is clear that impaired SIgA production and release can deeply impact on gut homeostasis, leading to decreased microbial diversity and mucosal dysbiosis [92]. Even though secretory IgM (SIgM) can be released into the gut lumen in response to IgA deficiency, this counteracting mechanism can only partially compensate such a defect. In particular, subjects with selective IgA deficiency exhibited alterations of microbial ecology with reduced alpha diversity [187,188]. Similar results have been reported in mice lacking *pIgR*, where SIgA levels were decreased at the mucosal surfaces due to impaired transcytosis across epithelial cells [93,94]. Again, microbial community was significantly altered in these mice, though compensatory mechanisms occurred to limit potential detrimental effects (e.g., increase of serum IgA levels and B cells, and increase of the frequency of macrophages, dendritic cells, and intraepithelial lymphocytes) [189,190,191,192].

## 3. Discussion and Therapeutic Perspectives

The maintenance of the intestinal barrier integrity and functions requires a fine-tuned balance among different specialized cells in order to ensure the physiological and protective crosstalk between intestinal microbes and host immune response, protection against xenobiotic substances, as well as nutrient absorption.

Nowadays, much effort has been made to explore the mechanisms underlying an altered intestinal barrier and gut dysbiosis in subjects carrying genetic polymorphisms or exposed to infections, medications and specific diets. In this regard, the recent advent of integrative multi-omic analyses has become a valid and promising means to better characterize the pivotal contribution of genetic defects and environmental factors in the impairment of gut homeostasis. For instance, metataxonomic and metagenomic data have helped to characterize microbiota profiles in different pathological conditions (such as IBD) and, in addition, highly contributed to uncover the dynamics and functional interactions among bacteria, metabolite pools, and host genetics [193,194,195]. Similarly, transcriptomic, proteomic, and metabolomic techniques have strongly helped to better define the impact of gut dysbiosis in IBD pathogenesis as well as in the development and progression of other chronic diseases [196,197,198,199,200]. Altogether, these approaches have definitely provided important knowledge to predict the onset and progression of pathological conditions, as well as their response to treatments, thus representing a key tool for the development of new diagnostic, prognostic, and therapeutic strategies [197,198,201,202,203].

Restoring the intestinal barrier physiological functions following a perturbation has always been considered a fascinating and promising approach to treat chronic inflammatory diseases such as IBD, and, indeed, multiple attempts have been made in this direction.

Over the past decades, it was found that flavonoids, which are phytochemicals with biological activity ubiquitously distributed in edible plants, can directly treat IBD through various mechanisms, including anti-inflammatory and antioxidant actions, which preserve the epithelial barrier; immunomodulatory functions in the intestine, which shape the composition and function of the microbiota; and the modulation of specific enterohormones (such as glucagon-like peptide 1 and dipeptidyl peptidase-4 inhibitors) [204,205,206]. Taken together, these observations suggest that the maintenance of gut homeostasis can be modulated by such bioactive compounds. Further studies on the basic functional role of flavonoids in IBD could contribute to establish new effective therapeutic options for the treatment of this disease in the future.

Accumulating evidence has shown the importance of the JAK-STAT signaling pathway in the pathogenesis of IBD by inducing, both directly and via the modulation of inflammatory cytokines, changes in intestinal paracellular permeability through the regulation of tight junction protein expression and localization. Given that, inhibitors of the JAK-STAT pathway are currently a therapeutic option for IBD patients. However, these compounds may present potential risks, including non-specificity and toxicity [207].

As decreased microbial diversity, overgrowth of pathogens, and uncontrolled immune/inflammatory response characterizing IBD were seen to further sustain defective barrier function (although most often these events are just a direct consequence of barrier impairment), other curative possibilities aim at treating these aspects.

In this context, change in dietary habits is one of the most powerful ways to alter the gut microbiome [208,209]. For instance, high-fiber diets (e.g., vegetarian, vegan, Mediterranean), which are low in red meat and higher in unsaturated fatty acids, are associated with a more beneficial microbiome composition, an increased microbial diversity, and more health-promoting bacteria (e.g., *Bifidobacteria*, *Lactobacillus*), as well as higher levels of small chain fatty acids (especially butyrate) [210,211,212,213]. These modulations result in a more thick mucus layer and an improved function of the intestinal barrier. Nevertheless, although dietary interventions aimed at reducing inflammatory chronic diseases and improving the microbiome are promising, the field is still evolving and, due to a large heterogeneity of the studies, drawing definitive conclusions has proved difficult in the past.

Finally, it should be mentioned that some IBD medications (e.g., steroids, aminosalicylates, and anti-TNF-α agents), as well as the use of probiotics/prebiotics have been reported to positively affect either the composition/metabolism of gut microbiota or the metabolic status of intestinal cells by altering the intestinal biota [214]. These drugs/strategies may thus represent valid therapeutic options.

A different and more fascinating approach is aimed at targeting the primary genetic defects undermining the intestinal barrier homeostasis with the delivery of therapeutic genetic information through viral vectors (e.g., lentiviruses, adenoviruses). However, it is worth underlining that, in this regard, no therapies have been validated in clinical studies so far, due to a number of drawbacks. For instance, the viral access to transduce intestinal epithelial cells with the gene of interest can be damped by the mucosal barrier itself as the mucus layer and/or the tight junctions may interfere with this process [215,216,217,218,219,220,221].

In addition, as enterocytes are characterized by a high turnover, this precludes their long-term transduction [222,223]. Finally, multiple treatments with viral vectors may trigger unwanted host immune responses that could compromise the efficacy of this therapeutic approach [224].

## 4. Conclusions

In conclusion, targeting intestinal barrier dysfunctions, and in particular those related to primary genetic defects, represents a very interesting and challenging approach to treat gut-related diseases. However, further efforts are necessary to transfer experimental findings on this complex topic into clinical practice.

## Figures and Tables

**Table 1:** Intestinal barrier alterations and related pathological conditions.

Disease	Observation	Ref.
**IBD**	Impaired mucus production and secretion Altered expression and distribution of epithelial junctional complexes Increased intestinal permeability and bacterial translocation Modulation of intestinal permeability by inflammatory cytokines	[15,16,17,18,19]
**Celiac disease**	Gliadin-induced zonulin secretion and increased intestinal permeability by a MyD88-dependent mechanism mediated by the CXCR3 receptor Altered expression and distribution of epithelial junctional complexes Inflammatory cytokine-driven impairment of tight junction assembly and distribution	[20,21,22,23,24]
**CDI**	Antibiotic therapy reduces colonization resistance against *Clostridioides difficile* by altering the microbiota-related protective barrier, as well as the microbial metabolism in the intestine	[25,26,27]
**IBS**	Increased intestinal permeability and molecular alterations in the tight junction expression and signalling pathways Correction of visceral hypersensitivity and pain by restoration of barrier dysfunction	[28,29,30,31,32,33,34]
**CRC**	Dysregulated expression of junctional complexes induces altered intestinal permeability and contributes to tumorigenesis and colonic epithelial cell invasiveness Gut dysbiosis resulting from altered intestinal permeability triggers and sustains chronic inflammation and genotoxic stress	[35,36,37,38,39,40]
**Obesity**	High-fat diet triggers gut dysbiosis and increases intestinal permeability in obese individuals Hyperglycemia negatively impacts on the expression and integrity of epithelial junctional complexes	[41,42,43,44]
**Type 1 diabetes**	Increased intestinal permeability can precede disease development Zonulin upregulation modulates the expression of epithelial junctional complexes and associates with increased gut permeability in subjects with type 1 diabetes and their relatives Restoration of barrier function can prevent diabetes development in disease-prone animals Hyperglycemia increases intestinal barrier permeability by altering tight and adherence junction integrity	[44,45,46,47,48,49,50]

Abbreviations: IBD: Inflammatory Bowel Diseases; MyD88: Myeloid differentiation primary response 88; CXCR3: C-X-C Motif Chemokine Receptor 3; CDI: *Clostridioides difficile* infection; IBS: Irritable Bowel Syndrome; CRC: Colorectal cancer.

**Table 2:** Genetic defects affecting intestinal barrier homeostasis.

Category	Gene	Effects on Intestinal Barrier	Ref.
Cell commitment	*Hes1*	Reduced production of AMPs and mucus, gut dysbiosis, and inflammation. Precocious differentiation of Paneth cells. Impaired specification of IECs into enterocytes.	[51,52,53]
	*Math1*	Decreased frequency of goblet cells.	[54]
	*Stk11*	Impaired released of AMPs and IL-18. Colitogenic bacteria overgrowth.	[55]
	*Cdx2*	Altered mucus production and increased intestinal permeability and susceptibility to DSS-induced colitis.	[15,56]
	*Gata6*	Impaired stem cell proliferation, reduction in villus length, Paneth cell, and enterocyte and enteroendocrine cell frequency. Increased number of goblet-like cells. Decreased levels of ZO-1 and increased intestinal permeability and susceptibility to experimental colitis and ileitis. Gut dysbiosis.	[57,58,59]
	*Sox9*	Lack of differentiated Paneth cells, crypt enlargement, gut dysbiosis.	[60,61]
Junctional Complexes	*Jam-A*/*F11R*	Increased intestinal permeability, low-grade intestinal inflammation, and increased susceptibility to DSS-induced colitis.	[62]
	*Cldn-2* and *Cldn-15* double-KO	Impaired paracellular Na^+^ flow and malnutrition.	[63]
	*Tjp1*	Apical surface brush border membrane and crevasses at intercellular junctions between enterocytes. Increased susceptibility to experimental colitis, delayed cell division, and mucosal healing.	[64,65]
	*P* *tpn2*	Increased claudin-2 expression, intestinal permeability, and inflammatory cytokine production.	[66]
Mucus layer	*Muc2*	ER stress and decreased frequency of goblet cells, altered mucus production, increased intestinal permeability, gut dysbiosis, and chronic intestinal inflammation.	[16,67,68]
	*Gfi1*	Accumulation of secretory progenitors, decrease in mucus and AMPs release.	[69]
	*Foxo1*	Impaired mucus layer formation, overgrowth of mucin-degrading bacteria, and decrease of short-chain fatty acid-producing microbial species. Enhanced susceptibility to infection and inflammation.	[70]
Paneth cells	*Nod2*	Impaired α-defensins secretion.	[71,72]
	*Atg16l1*	Impaired autophagy in response to viral infection and decreased AMPs release.	[73,74]
	*Xbp1*	Chronic ER stress in response to viral infection and decreased AMP release.	[75]
	*Tcf4*	Reduced α-defensins secretion and CD development.	[76,77]
PRRs	*MyD88*	Increased stem cell proliferation.	[78]
	*Nod2*	Impaired pathogen sensing and clearance, and gut dysbiosis. No protection against oxidative stress-mediated cell death, and impaired epithelial regeneration.	[79,80,81,82,83]
Oxidative Burst	*Duox2*	Enhanced pathogen translocation to host lymphatic tissues.	[84]
	*Cyba*	Decreased DUOX2 activity in response to *Citrobacter rodentium*.	[85]
Xenobiotic Receptors	*Pxr-Nr1I2*	Dysregulated TLR4-NF-κB signalling pathway, reduced ZO-1 and E-cadherin expression, increase in Claudin-2 levels. Higher susceptibility to IBD development.	[86,87,88]
	*AhR*	Increased susceptibility to intestinal infection (*Citrobacter rodentium*), reduced mucus production, impaired tight junctions, and crypt hyperplasia.	[89,90,91]
Secretory IgA	*IgA*	Gut dysbiosis.	[92]
	*pIgR*	Impaired SIgA transcytosis across epithelial cells and gut dysbiosis.	[93,94]

Abbreviations: Hes1: Hairy and enhancer of split 1; Math1: Mouse atonal homolog 1; Stk11: Serine threonine kinase 11; IL-18: Interleukin-18; Cdx2: Caudal type homeobox 2; DSS: Dextran Sodium Sulfate; Gata6: GATA binding factor 6; ZO1: Zonula Occludens-1; Sox9: SRY-Box Transcription Factor 9; Jam-A/F11R: Junctional adhesion molecules/F11 receptor; Cldn: claudin; Tjp1: Tight junction protein-1; Ptpn2: Protein tyrosine phosphatase non-receptor type 2; Muc2 Mucin-2; ER: Endoplasmic Reticulum; Gfi1: Growth factor independent 1; Foxo1: Forkhead box protein O1; Nod2: Nucleotide Binding Oligomerization Domain Containing 2; Atg16l1: Autophagy Related 16 Like 1; Xbp1: X-Box Binding Protein 1; Tcf4: transcription factor 4; CD: Crohn’s disease; MyD88: Myeloid differentiation primary response 88; Duox2: Dual oxidase 2; Cyba: Cytochrome B-245 Alpha Chain; Pxr/Nr1I2: Pregnane X receptor/Nuclear receptor subfamily 1 group I member 2; TLR4: Toll-like receptor-4; NF-kB: nuclear factor kappa-light-chain-enhancer of activated B cells; AhR: Aryl Hydrocarbon Receptor; IgA: Immunoglobulin A; pIgR: poly immunoglobulin receptor; AMP: Antimicrobial peptides; ZO-1: Zonula Occludens-1; ER: Endoplasmic reticulum; SIgA: Secretory immunoglobulin A.

**Table 3:** Environmental factors affecting intestinal barrier homeostasis.

**Category**	**Diet**	**Effects on Intestinal Barrier**	**Ref.**
Junctional Complexes	Gluten	Alterations in adherent junctions and desmosomes and increased intestinal permeability and susceptibility to experimental colitis. Disassembly of ZO-1 from the tight junctional complex.	[20,95]
	Glucose/Fructose	TJ and AJ proteins dysfunction, increased susceptibility to pathogen infection, gut dysbiosis, metabolic syndrome, oxidative stress, and chronic inflammation	[44,96]
	High-fat diet	ER stress in IECs, impairment of Claudin-1 expression and mucus barrier, and increased endotoxin serum levels. Increased taurocholic bile acid production and gut dysbiosis.	[97,98]
	Ethanol	Altered ZO-1 and occludin localization and impaired paracellular permeability.	[99,100]
Mucus Layer	Fiber-deprived diet	Thinner mucus layer, gut dysbiosis, and chronic intestinal inflammation.	[67,101]
Paneth cells	Vitamin D deficiency and exposure to high-fat diet	Impaired expression of α-defensins, MMP7, and tight junction-related proteins; increased intestinal permeability; gut dysbiosis; and metabolic syndrome. Indiction of ER stress and secretion of misfolded α-defensins.	[102,103]
	High fat diet	Decreased AMP expression, ER stress and autophagy induction, and gut dysbiosis.	[104]
	Western diet (deoxycholic acid)	Excessive activation of the farnesoid X receptor and type I interferon signalling pathways.	[105]
Xenobiotic receptors	AhR ligand-free diet	Higher susceptibility to experimental colitis and gut dysbiosis.	[106,107]
**Category**	**Bacterial infection**	**Effects on intestinal barrier**	**Ref.**
Junctional Complexes	Infection by *Salmonella typhimurium*	Increased claudin-2 expression and bacterial invasion.	[108]
	Infection by *Vibrio cholerae*	Zonula occludes toxin production and altered paracellular permeability.	[109,110]
PRRs	LPS	Increased intestinal permeability.	[111]
**Category**	**Medication exposure**	**Effects on intestinal barrier**	**Ref.**
Junctional Complexes	Antibiotic treatment	Decreased production of microbial-derived short-chain fatty acids. Gut dysbiosis. Reduced ZO-1, occludin, and claudin-1 expression, and increased intestinal permeability. Altered microvilli morphology and reduced rate of intestinal epithelial cell turnover.	[112,113]

Abbreviations: ZO-1: Zonula Occludens-1; TJ: Tight junction; AJ: Adherent junction; ER: Endoplasmic Reticulum; IECs: Intestinal Epithelial Cells; MMP: Matrix Metallopeptidase; AMP: Antimicrobial peptide; AhR: aryl hydrocarbon receptor; PRR: Pattern recognition receptors; LPS: lipopolysaccharide; IECs: intestinal epithelial cells.

## Data Availability

Not applicable.
